# EXPLORING MULTIDIMENSIONAL RESILIENCE IN OLDER VETERANS WITH PTSD DURING THE PANDEMIC

**DOI:** 10.1093/geroni/igac059.598

**Published:** 2022-12-20

**Authors:** Katherine Hall

**Affiliations:** Durham VA Health Care System, Durham, North Carolina, United States

## Abstract

The COVID-19 pandemic and restrictions for physical and social distancing has affected all older adults, but one segment that has unique needs and experiences is older veterans with PTSD. This presentation will explore how PTSD symptomology, and psychosocial functioning in this population have changed compared to pre-pandemic findings. The impact of the pandemic on daily life activities and functional impairment will also be explored. Participants recruited to a wellness clinical trial for older veterans with PTSD at two different timepoints (pre-pandemic, n=54; post-pandemic, n=28) completed PTSD and mental health assessments, and a physical performance battery testing strength, mobility, balance, and aerobic endurance. PTSD symptoms, specifically avoidance and hypervigilance were markedly lower in the post-pandemic sample. Higher levels of physical impairment were observed in the post-pandemic sample, suggesting a need for targeted outreach and health promotion programs among older adults with PTSD symptoms during the pandemic.

